# Homology Model and Docking-Based Virtual Screening for Ligands of Human Dyskerin as New Inhibitors of Telomerase for Cancer Treatment

**DOI:** 10.3390/ijms19103216

**Published:** 2018-10-18

**Authors:** Romina Gabriela Armando, Diego Luis Mengual Gómez, Ezequiel Ivan Juritz, Pablo Lorenzano Menna, Daniel Eduardo Gomez

**Affiliations:** 1 Laboratory of Molecular Oncology, Department of Science and Technology, Quilmes National University, Buenos Aires B1876BXD, Argentina; romina.armando@unq.edu.ar (R.G.A.); diego.mengualgomez@unq.edu.ar (D.L.M.G.); 2 Center for Bioinformatics and Integrative Biology, Facultad de Ciencias de la Vida, Universidad Andrés Bello, Santiago 8370146, Chile; ejuritz@gmail.com; 3 Laboratory of Molecular Pharmacology, Department of Science and Technology, Quilmes National University, Buenos Aires B1876BXD, Argentina; plmenna@unq.edu.ar

**Keywords:** telomerase, DKC1, hTR, inhibitors, cancer

## Abstract

Immortality is one of the main features of cancer cells. Tumor cells have an unlimited replicative potential, principally due to the holoenzyme telomerase. Telomerase is composed mainly by dyskerin (DKC1), a catalytic retrotranscriptase (hTERT) and an RNA template (hTR). The aim of this work is to develop new inhibitors of telomerase, selecting the interaction between hTR–DKC1 as a target. We designed two models of the human protein DKC1: homology and ab initio. These models were evaluated by different procedures, revealing that the homology model parameters were the most accurate. We selected two hydrophobic pockets contained in the PUA (pseudouridine synthase and archaeosine transglycosylase) domain, using structural and stability analysis. We carried out a docking-based virtual screen on these pockets, using the reported mutation K314 as the center of the docking. The hDKC1 model was tested against a library of 450,000 drug-like molecules. We selected the first 10 molecules that showed the highest affinity values to test their inhibitory activity on the cell line MDA MB 231 (Monroe Dunaway Anderson Metastasis Breast cancer 231), obtaining three compounds that showed inhibitory effect. These results allowed us to validate our design and set the basis to continue with the study of telomerase inhibitors for cancer treatment.

## 1. Introduction

The term “cancer” represents a large group of more than one hundred diseases that can affect almost any structure of an organism. Cancer is characterized as a complex disease involving different factors. Malignant tumors share certain biological features, including: sustained proliferative ability; lack of sensitivity to inhibitory growth signals; resistance to cell death; ability to induce angiogenesis; activation of invasion and metastasis; and unlimited replicative immortality [1].

In more than 85% of malignant tumors, immortality of cancer cells is mainly due to the activity of the holoenzyme telomerase, which is not expressed in most somatic cells. The active human telomerase is a ribonucleoprotein, which is mainly composed of a catalytic subunit called hTERT (human telomerase reverse transcriptase) and a template RNA called hTR (human telomerase RNA) [2,3]. However, several other proteins are necessary for in vivo assembly, subcellular trafficking and telomere association of the functional telomerase holoenzyme. Among these proteins, we should highlight the role of dyskerin pseudouridine synthase (DKC1), which allows the correct assembly and stabilization of mature hTR, which is essential for telomerase activity [4,5].

DKC1 is an evolutionarily conserved protein of 58 kDa that plays an active role in telomerase stabilization and maintenance, as well as recognition of snoRNAs (small nucleolar RNAs) containing H/ACA sequences (Motif H box/ ACA box sequences); a type of RNA that provides stability during biogenesis and assembly of H/ACA small nucleolar ribonucleoproteins (snoRNPs). Also, DKC1 may play additional roles in nucleo-cytoplasmic shuttling, the DNA damage response, and cell adhesion. Its sequence presents a high degree of phylogenetic conservation, evidencing its biological relevance. However, at present, there is no crystal structure available of human DKC1 (hDKC1). This protein is divided into at least three well preserved functional domains: the DKC-like domain (DKCLD; 48–106 amino acids (aa), typical of this protein family but still of unknown function; the TruB_N pseudouridine synthase catalytic domain (110–226 aa), involved in the pseudouridylation process; and finally, the PUA (pseudouridine synthase and archaeosine transglycosylase) RNA binding domain (297–370 aa), which is involved in the recognition of H/ACA RNAs, such as hTR. In addition, four low complexity regions (aa 11–20, 421–455, 467–480 and 498–507), rich in lysine and arginine, are identified within the putative nuclear localization signals (NLSs) [6].

The study of DKC1 and its pathological role was originated several years ago due to the presence of irregularities on the structure or the expression level of this protein in different diseases. The most important one is dyskeratosis congenita (DC), a bone marrow failure syndrome where its clinical presentation exposes a variety of symptoms characterized by the classic triad of mucocutaneous features, followed by a large number of additional symptoms such as bone marrow failure, defective stem cells, premature aging and increased tumor susceptibility [7].

Interest in DC has largely focused on the fact that some families with this disease have mutations in the gene encoding the RNA component of telomerase. However, the most widely recognized form of this disease is caused by mutations in hDKC1. These mutations are also related to Hoyeraal–Hreidarsson syndrome; characterized by developmental delay, immunodeficiency, aplastic anemia and early mortality, this syndrome is currently considered a severe variant of DC. It is essential to highlight that these diseases are characterized by the presence of short telomeres and dysfunction of telomerase activity, supporting the fundamental role of this protein in telomere maintenance [8].

As mentioned above, several mutations associated with these diseases have been reported along the coding sequence of hDKC1. Interestingly, a vast majority are located in the PUA domain. Among them, the mutation positioned in the aa A353 is responsible for more of 40% of DC cases. Another reported mutation is in the aa K314; it causes an isoform of hDKC1 that is associated with the most severe variants of DC. Evidence suggests that these kind of mutations could be affecting the binding of substrate RNAs [5,9].

Considering that the vast majority of cancer types rely on the holoenzyme telomerase for tumor progression, and that hDKC1 is highly relevant in telomere maintenance, this protein is an interesting target for the development of anti-cancer therapies [10]. This work aims to study a novel strategy for the rational development of new inhibitors of the assembly of the holoenzyme telomerase, based on the interruption of the interaction of hDKC1 with hTR. By in silico approaches, we obtained two models of hDKC1 with suitable characteristics for the search of candidate compounds by docking-based virtual screening (DBVS), achieving novel drugs with inhibitory effects on telomerase activity and potential clinical use for cancer treatment.

## 2. Results

In order to identify the most valuable site for the development of the new inhibitors, we performed a number of analyses in silico.

### 2.1. Physicochemical Properties and the Abundance of aa in hDKC1 Protein

**Figure 1:**
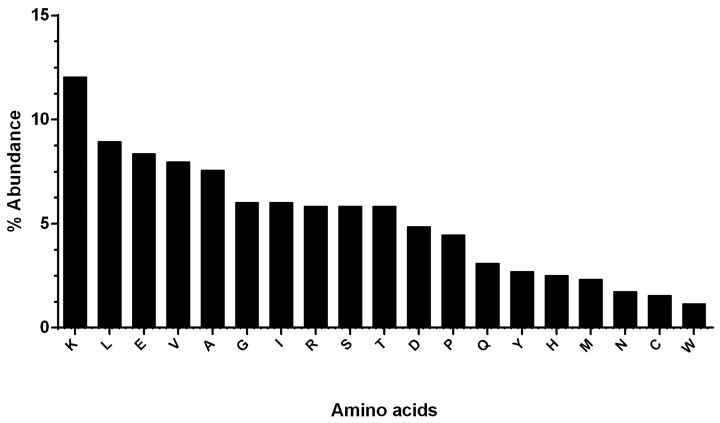
Graphical representation of the abundance of the 20 amino acids (aa) present in human dyskerin pseudouridine synthase (hDKC1). Lysine has the highest abundance, and phenylalanine has the lowest abundance.

Results obtained by the use of ExPASy (Expert Protein Analysis System) showed that the hDKC1 protein sequence has 514 aa residues, resulting in a protein with an average molecular weight of 57.6 kDa. Furthermore, the most abundant aa in hDKC1 is lysine, with 12.06%, followed by leucine, glutamate and valine (8.95%, 8.37% and 7.98%, respectively). Phenylalanine and tryptophan were among the lowest abundant residues, with 0.97% and 1.17% respectively, followed by cysteine, asparagine and methionine (Figure 1).

The physicochemical parameters predicted a positively charged protein as a result of the high number of positively charged residues (arginine 5.84% and lysine 12.06%) in contrast with negatively charged ones (aspartic acid 4.86% and glutamic acid 8.37%).

The atomic composition of hDKC1 is 8255, with 2553 carbon (C), 4209 hydrogen (H), 723 nitrogen (N), 750 oxygen (O) and 20 sulfur (S). Furthermore, the protein is basic, with an isoelectric point of 9.46. The estimated half-life of this protein showed that it can remain intact without being degraded for 30 h in humans, less than 20 h in yeast and less than 10 h in *E. coli*, and its extinction coefficient is 54,360 M^−1^·cm^−1^. Finally, the generated aliphatic index was 89.14, with −0.483 GRAVY (grand average of hydropathicity) and an instability index of 44.94.

### 2.2. Prediction of the Two-Dimensional Structure of hDKC1

**Figure 2:**
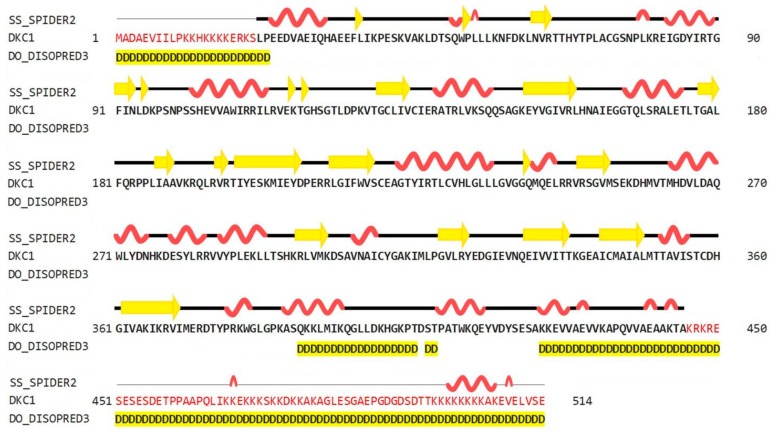
Secondary structure prediction of hDKC1 using HHPred (SPIDER2 (Scoring Protein Interaction Decoys using Exposed Residues) for secondary structure prediction, and DISOPRED (Disorder Prediction Server) for unstructured or disordered region predictions). Yellow arrows represent beta sheets; alpha helixes are shown in red; disordered regions are highlighted in yellow.

As shown in Figure 2, the predictions obtained using the HHPred server (Homology detection by Hidden Markov modes Prediction) revealed that the secondary structure of hDKC1 contains 26.85% α-helices, 10.51% β-strands, and 62.65% loops. Both N- and C-terminals showed a disorganized structure. Regarding this feature, it was reported that the unique function of residues 1 to 21 is to act synergistically with the C-terminal nuclear localization sequence (NLS) to ensure dyskerin nucleolar localization. Likewise, the biological relevance of the C-terminal region (390–514 aa) is supported by the presence of a bipartite NLS and of several potentially modified residues [6]. These observations could explain the unstructured organization of this region, which is highlighted in yellow in Figure 2.

### 2.3. Sequence and Secondary Structure Analysis between hDKC1 and Saccharomyces Cerevisiae Dyskerin

**Figure 3:**
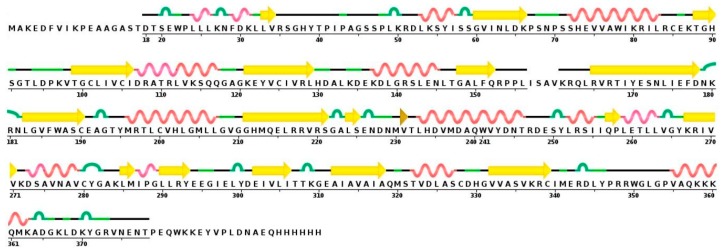
Sequence and secondary structure of *S. cerevisiae* dyskerin obtained from the 3UAI Protein Data Bank (PDB) file. Yellow arrows represent beta sheets; alpha helixes are shown in red; turns are colored in green.

As mentioned above, there is no crystal structure available for hDKC1, but it has a highly evolutionary conserved sequence. From all reported dyskerins in the Protein Data Bank (PDB), our studies showed that the highest value of sequence homology was present in the structures 3UAI and 3U28 belonging to *S. cerevisiae*. In comparison, the 3UAI structure showed more structural information than the 3U28 structure (data not shown). Taking advantage of this fact, we decided to generate a three-dimensional (3D) structure model of hDKC1 by homology with *S. cerevisiae* dyskerin (chain A from 3UAI). The initial step consisted of an analysis between the predicted secondary structure of hDKC1 and the secondary structure obtained from 3UAI. As presented in Figure 3, neither C- nor N-terminal sequences are included in the crystal structure of 3UAI. This correlates with the results observed in Figure 2, where N- and C-terminal sequences had no secondary structure and they were reported as cellular localization sequences. Based on these observations, we decided to model the sequence of hDKC1 comprising the residues from position 22 to 420, where a secondary structure was shown.

### 2.4. Predicted 3D Homology Model of hDKC1 by I-TASSER

**Figure 4:**
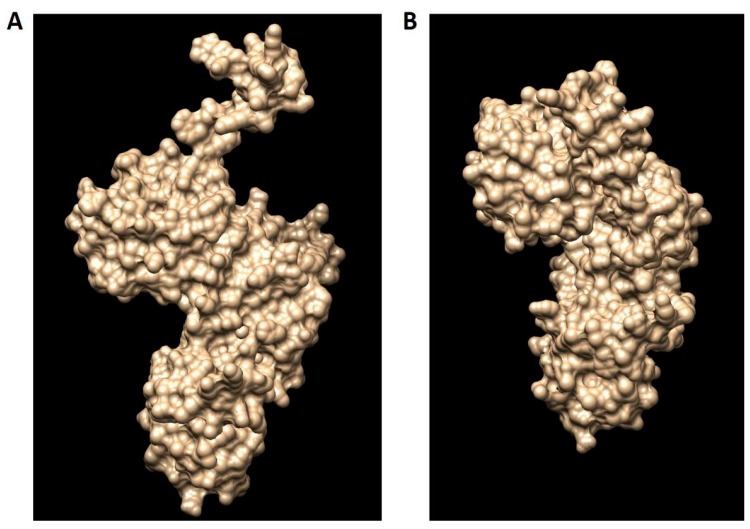
The hDKC1 models obtained by I-TASSER (Iterative Threading Assembly Refinement). (**A**) hDKC1 homology model; (**B**) hDKC1 ab initio model.

Using I-TASSER (Iterative Threading Assembly Refinement), the 3D model structure of hDKC1 was carried out by two different strategies: the first one consisted of using the structure of 3UAI as template for modelling the hDKC1 sequence by homology. The second one was an ab initio model, where the software builds the 3D structure based on energy calculus. Both models are shown in Figure 4, visualized using MGLTools (Molecular Graphics Laboratory Tools).

I-TASSER evaluates the model using two parameters. The first one is the C-score, which is the confidence score to evaluate the quality of a predicted model. The C-score is typically in the range of −5–2, where a C-score of higher value indicates a model with a high confidence and vice-versa. Another important parameter to take into account is the TM-score (Template Modeling score), which is a proposed scale for measuring the structural similarity between two structures. A TM-score of > 0.5 indicates a model of correct topology and a TM-score < 0.17 indicates a random similarity [11]. As shown in Table 1, the C-score for both models is adequate, being the homology model the most confident one. Although the TM-score and RMSD (Root-Mean-Square Deviation) values of both models are acceptable for a proper design, the homology one showed more robust results and was chosen for our analysis.

### 2.5. 3D Structure Validation

**Figure 5:**
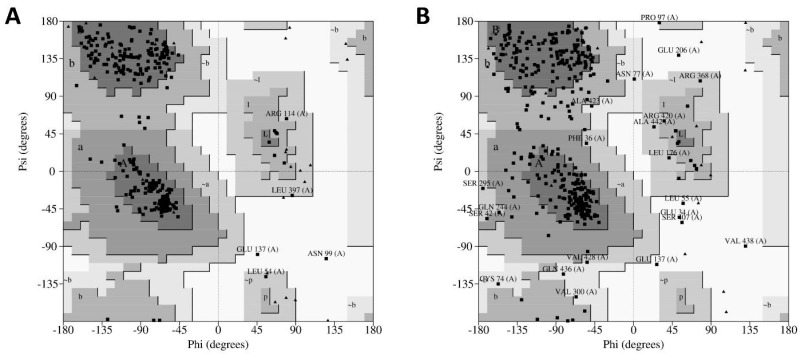
PROCHECK (Program to check the stereochemical quality of protein structures) results of modelled hDKC1 using the generated models from I-TASSER. (**A**) Ramachandran plot for hDKC1 homology model; (**B**) Ramachandran plot for hDKC1 ab initio model. Residues in most favored regions (A, B, L), residues in additional allowed regions (a, b, l, p) and residues in generously allowed regions (~a, ~b ~l, ~p).

Subsequently, the quality of models was validated using PROCHECK (Program to check the stereochemical quality of protein structures), a program that relies on Ramachandran plots for structure verification. According to this software, a good quality model could be expected to have over 90% of its residues in the most favored region [12]. As shown in Figure 5A, results from PROCHECK determined that the homology model has 92.1% of its residues in the most favored regions, 6.3% in the additional allowed regions, 0.9% in the generously allowed regions and 0.6% in the disallowed regions. In contrast, results from the ab initio model showed 79.1% of residues in the most favored regions, 18.8% in the additional allowed regions, 3.7% in the generously allowed regions and 1.6% in the disallowed regions (Figure 5B). Therefore, regarding the percentage distribution of the residues, the homology model has a higher quality than the ab initio model.

Additionally, the G-factor for each model was determined using the same software. This value provides a measure of how unusual a determination of a given stereochemical property is using this program. A G-factor of less than −0.5 is unusual and less than −1.0 is highly unusual [12]. The G-factor obtained for the homology model was −0.27 for dihedral angles, −0.02 for main chain covalent forces and −0.16 overall. Conversely, the values determined for the ab initio model were −0.92, −0.09 and −0.57, respectively. This evidence strongly suggests that the homology model has more accurate features than the ab initio model. Consequently, we continued this work using the hDKC1 homology model, since it was demonstrated to be a more robust model in all the parameters analyzed.

### 2.6. Study of Mutation Stability

**Figure 6:**
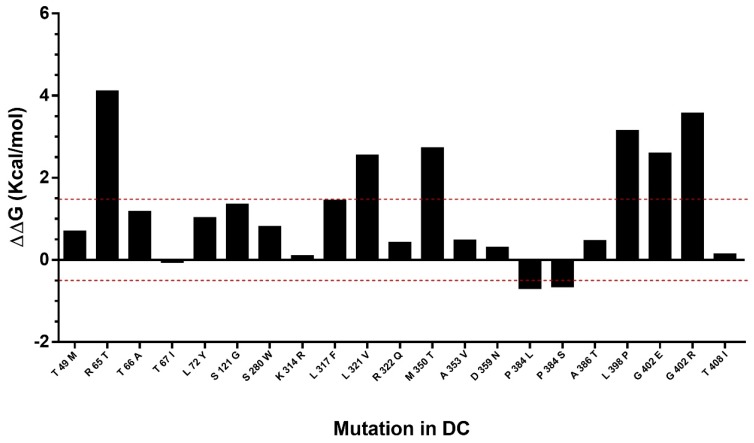
Analysis of mutation stability in the hDKC1 model by Foldx software. Mutations comprised between red dashes lines (−0.5 Kcal/mol to 1.5 Kcal/mol) correlates with the mutations that are not destabilizing. DC = dyskeratosis congenita.

The effect of mutations described in DC were predicted in the hDKC1 model. A comparison between the stability of all the reported mutations vs. the wild type protein was made using the Foldx suite. The predicted ΔΔG values of the mutations fell in a range of −0.7 to 4.1 kcal/mol (Figure 6). This analysis led us to the identification of eight destabilizing mutations, since the mutations with ΔΔG values under −0.5 or over 1.5 kcal/mol could significantly reduce protein stability [13].

### 2.7. Evaluation and Recognition of Hydrophobic Pockets on hDKC1 Model

By using the FPocket software, we searched and analyzed all the hydrophobic pockets present in our model, with the aim of determining the best ones in order to set the parameters for DBVS. As a result, we found 21 pockets, ordered by a relevance parameter determined by the following formula: (pocket score/the highest score) × 100. The pockets, their relevance parameter, volume and the involved residues are presented in Table 2.

### 2.8. Study and Determination of the Hydrophobic Pocket Containing the Mutation K314

**Figure 7:**
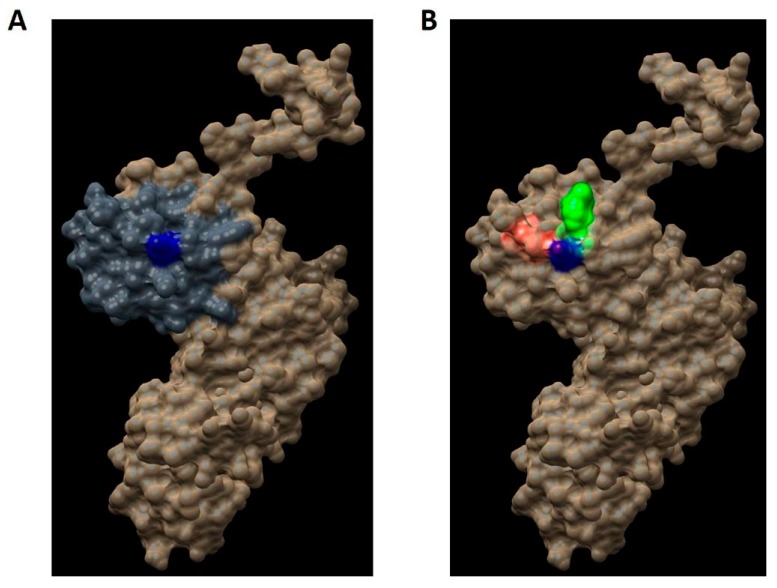
Regions in the hDKC1 three-dimensional (3D) structure model. hDKC1 is colored in beige. Residue K314 is indicated in blue (**A**) The PUA domain is colored in dark gray (**B**) Pocket 2 and Pocket 13 are highlighted in red and green, respectively.

As we mentioned in the introduction, the aim of this work is to design a novel strategy for the rational development of new inhibitors of the assembly of the holoenzyme telomerase, based on the interruption of the interaction of hDKC1 with hTR. To carry out this work, we decided to emulate the phenotype showed and reported in DC, where a mutation on the residue K314 in the hDKC1 sequence leads to a loss in telomerase function caused by the inability of hDKC1 to join hTR. As shown in Figure 6, the K314R site mutation exhibits a ΔΔG value of 0.104 kcal/mol, implying the change does not reduce protein stability and thus it is an accurate site to direct the DBVS (Docking Based Virtual Screening). Consequently, from all the pockets found, we decided to work with those which contain the residue K314. Two pockets containing this residue were found: Pocket 2 and Pocket 13. Both are included in the PUA domain and are worthy of consideration due to their proper values of relevance, according to FPocket analysis. Figure 7 shows the DKC1 model, the PUA domain and the two pockets containing residue K314.

### 2.9. Docking Based Virtual Screening on the hDKC1 Model by AutoDock Vina

Once the 3D structure model of hDKC1 was validated and the target pocket was defined, we set the parameters to carry out the DBVS using AutoDock Vina Software. We established the alpha carbon on residue K314 as the docking center. We defined a box of 25 Å to cover the two pockets described above. Finally, we decided to use the library “Advanced Collection from Enamine”, since it contains more than 400,000 compounds suitable for the development of new drugs for clinical use.

As result of DBVS, a ranking of candidate compounds was obtained. These compounds are ordered by docking energy, being better candidates those with more negative values. The first 10 candidates obtained from the DBVS are listed in Table 3.

### 2.10. In Vitro Screening of the Candidate Compounds by Telomerase Activity Assay

**Figure 8:**
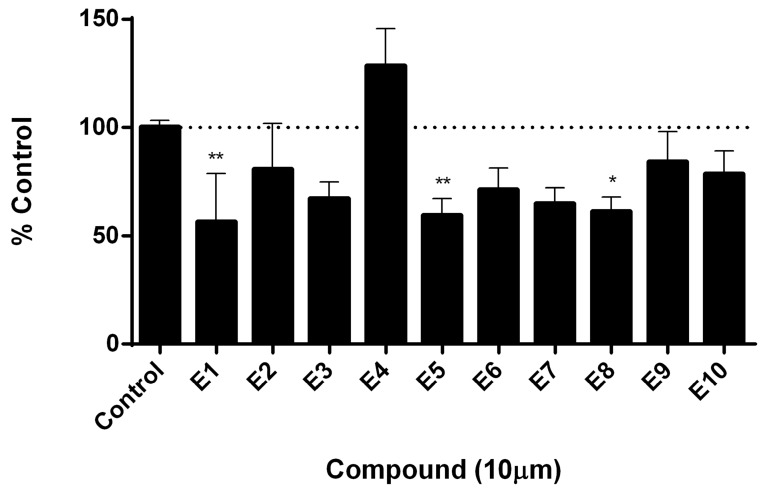
Determination of telomerase activity by qPCR. Quantification of telomerase activity was carried out by real time PCR with specific primers, using a protein extract from treated and untreated telomerase positive cells as a template for 48 h. Dashes line indicates the 100% of the control. Values represent media ± SEM; *n* = 6, * *p* < 0.5 ** *p* < 0.01 vs. control (ANOVA followed by Dunnett).

With the aim of determining if the candidate compounds show the desired inhibitory effect, we performed an assay for measuring telomerase activity. This assay was carried out in a tumor cell line positive for telomerase activity. Results presented in Figure 8 show that at least three compounds produced a significant decrease in telomerase activity after treatment, reaching an inhibitory effect of almost 40%. This evidence supports the rational design proposed in this work and sets the basis to continue investigating these compounds as potential anti-tumor drugs.

## 3. Discussion

Nowadays, drug design is increasingly reliant on computer modeling techniques. This type of strategy is often referred to as computer-aided drug design. More specifically, drug design that relies on the knowledge of the three-dimensional structure of the biomolecular target is known as structure-based drug design. In order to generate this type of drug design, an increasingly important number of computational methods for improving the affinity, selectivity and stability of these protein-based therapeutics have also been developed [14,15,16].

Regarding anti-tumor therapies, although effective cytotoxic compounds have been identified, treatments directed to a specific target still have ample room for improvement. Taking into account the experience of our group in the study of telomerase and our expertise on drug design using computational and molecular biology tools [17], we decided to carry out a DBVS on hDKC1, with the aim of generating new compounds with inhibitory effect on telomerase activity for cancer treatment.

The basis for performing a DBVS is the availability of the crystallized structure of the target protein. There are several studies in which the crystallized structure of dyskerin is used to explain, in a very interesting way, the interactions and processes in which it is involved. However, the authors utilized the structures coming from yeast [18,19] or archeas [20,21]. Although these structures are useful for this kind of study, the aim of our work is directed towards the development of new drugs, therefore each step must be as accurate as possible in order to avoid the appearance of hurdles along the way. Due to the fact that hDKC1 has not yet been crystallized, the need to obtain a model as close as possible to its actual structure became one of the most relevant steps of this work. To carry out this goal, among the different bioinformatic tools in existence, we selected I-TASSER which has demonstrated significant advantages in protein structure prediction, combining various techniques from threading, ab initio modeling and atomic-level structure refinement approaches [22]. Furthermore, the Critical Assessment of protein Structure Prediction (CASP), a community-wide, worldwide experiment for protein structure prediction taking place every two years since 1994 [23], has ranked I-TASSER as the best method for automated protein structure prediction in many CASP experiments [24]. For example, in the field of cancer research, it has been used to model the RAB38 protein (Ras related protein 38), relevant in melanoma [25] and RASSF2 (Ras Association Domain Family Member 2), studied in different tumor types [26]. Similarly, in the study of malaria, a model of M17LAP (M17- Family Leucine Aminopeptidase) was obtained and then used as a target protein in a DBVS assay [27]. In the same way, the structure by homology of Sortase A, a protein involved in listeriosis disease, was achieved [28].

As mentioned above, we decided to use the hDKC1 sequence corresponding to aa 21 to 420, since the first 20 aa and the last 96 aa were reported as signaling peptides with few relevant mutations [6] and, as observed in the secondary structure analysis, they correlated with unstructured areas (Figure 2 and Figure 3). From this sequence, and using the crystallized structure of the *S. cerevisae* dyskerin (3UAI) as a template, we performed both ab initio and homology modeling of hDKC1 using I-TASSER. It is important to highlight that yeast dyskerin is crystallized together with SHQ1 (H/ACA ribonucleoprotein assembly factor), NOP10 (Nucleolar protein 10) and GAR1 (H/ACA ribonucleoprotein complex subunit 1), which are proteins of the telomerase complex [18]. However, far from being a problem, this provides more accurate information on the real conformation of the protein in a biological context. Both processes converged in two models that were later analyzed by PROCHECK software, in order to check the stereochemical quality of the protein structure and analyze its overall and residue-by-residue geometry. The obtained results indicated that homology modeling presented appropriate quality parameters (Figure 5) [12], with values similar to the ones reported in other very interesting works [29,30]. Given this, studies were continued with the homology model of hDKC1.

Once the hDKC1 structure was obtained and validated, we set the parameters regarding the DBVS assay. The choice of the pocket and the docking center were based on several points: concerning the pocket site, the PUA domain has been recurrently reported as the domain that contains most of the mutations related to DC. Furthermore, the interaction with hTR occurs through this domain, so it represents an important domain for telomerase function [5,31,32]. Within this domain, the residue K314 was determined as the center of the docking. The choice of this residue as the center is due to the fact that it is reported as one of the most relevant mutations associated with the most severe manifestation of DC and is related with the impediment to stabilize the hTR in the complex [9]. Moreover, the stability of the point mutations reported for DC was evaluated using the analysis carried out using FoldX [6]. The results obtained showed ΔΔG values lower than 0.5 for K314 (Figure 6), which allowed us to assume that the mutation reported for this aa generates a binding mutant suitable for the DBVS assay [13].

It is pertinent to emphasize that the reasoning behind the choice of this site is the search for a molecule capable of interacting with this residue of hDKC1, preventing the stabilization of hTR and therefore, the formation of the telomerase complex. From the evaluation of all these parameters, modeling with appropriate characteristics was obtained to carry out the DBVS assay, setting the residue K314 as the center.

Regarding the use of DBVS as a search strategy for the development of new drugs, several examples can be found in the literature. Among them we can highlight inhibitors of the NS3 (Nonstructural protein 3) helicase of Dengue virus [33]; inhibitors of RAC1 (Ras-related C3 botulinum toxin substrate 1) in highly aggressive breast cancer lines [34]; and inhibitors of human XPF (*Xeroderma pigmentosum*, complementation group F) endonuclease for combination treatment with chemotherapy [35]. In this work, the molecular docking was carried out using the AutoDock Vina software, employing the homology model of hDKC1 obtained from the I-TASSER predictions as a target. The compounds’ library employed was the “Enamine Advanced Collection” which contains more than 450,000 drug-like compounds having led-like properties, with a molecular weight of less than 350, suitable lipophilicity values (logP < 3) and relevant pharmacophores groups, such as carboxyls, primary amines and amides. All these parameters are important in the development of a new drug [36]. The Enamine library exhibits a large number and variety of compounds with useful characteristics for performing high throughput screening assays, containing, in some cases, 10 times more compounds than other libraries [37]. Furthermore, several studies have been carried out using the libraries provided by Enamine. For example, inhibitors of tyrosine kinases [38]; STAT3 (Signal transducer and activator of transcription 3) inhibitors [39]; and inhibitors of *Mycobacterium tuberculosis* synthase [40], among others. This information represents great support in the selection of this library for the development of our work, since it was reported in different works that concluded successfully.

The employment of these methods for the development of new drugs for the treatment of cancer is already reported in the literature. Regarding the use of the modeled structure of a protein for a DBVS assay, a correlation between the success in modeling by software such as I-TASSER and the relative success in the virtual screening process was demonstrated [41]. This suggests that the combination of docking test and advanced structural modeling methods is a valuable approach in the study of the development of new drugs by DBVS, especially when there is a lack of crystallized structures of target proteins, as in our work. In addition to this evidence, molecules with anti-tumor action that are the result of strategies similar to the one proposed here already exist. This is the case for the work done on the DNA-dependent protein kinase (DNA-PK), which is involved in the process of recombination of non-homologous DNA and, therefore, is a relevant target for cancer research. In this work, the authors generated a model by homology of this enzyme, which was used in a DBVS assay. Once the compounds were obtained, they evaluated the in vitro effect, obtaining an inhibition of the kinase activity and a decrease in the proliferative ability in a combined treatment with doxorubicin and cisplatin in breast and lung tumor lines [42]. Another case, which applies to cancer and other viral and cardiovascular pathologies, is the search for inhibitors of human concentrative nucleoside transporters (hCNTs). hCNTs are not crystallized, so the authors generated models of homology of different variants based on the similarity and conservation they maintain with those of *Vibrio cholerae*, which are crystallized. The models obtained were used to carry out a DBVS assay. Then, the compounds were evaluated, obtaining as a result a molecule that has 25 times greater inhibitory ability than the commercial inhibitor [43]. Therefore, as shown by other authors, the bioinformatic path that we applied here is able to select molecules with the activity mentioned in our hypothesis.

**Figure 9:**
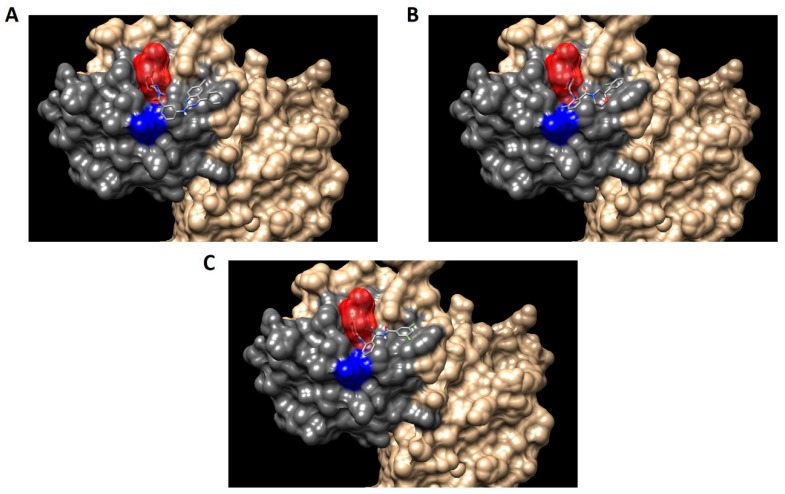
Spatial arrangement of the resulting compounds in the hDKC1 3D structure model. hDKC1 is colored in beige Residue K314 is indicated in blue, the PUA domain is colored in dark gray and Pocket 13 is highlighted in red. (**A**) E1; (**B**) E5; (**C**) E8.

Finally, the first 10 compounds derived from the DBVS assay were acquired (Table 3) and an in vitro screening was carried out by a telomerase activity assay on MDA MB 231 tumor cells (Figure 8). In summary, we found three compounds that exhibit telomerase inhibitory activity. Their spatial arrangement in the obtained model is shown in Figure 9. Although more research needs to be carried out, we have obtained results that validate our hypothesis, generating a basis to continue the study and development of inhibitors of telomerase activity that, for the first time, are aimed at the hTR–hDKC1 interaction, a crucial crossroad for the maintenance of telomerase activity in cancer cells.

## 4. Materials and Methods

### 4.1. Sequence Obtaining and Physicochemical Parameters Analysis of hDKC1

The aa sequence was obtained from the Universe Protein Resource (UniProt) (available online: http://www.uniprot.org/). The physicochemical parameters of the protein were generated from the ProtParam tool (available online: http://web.expasy.org/protparam/) using the ExPAsy server.

### 4.2. Secondary Structure Prediction

Prediction of the secondary structure of hDKC1 was done using the SPIDER2 secondary structure prediction server (available online: http://sparks-lab.org/server/SPIDER2). SPIDER2 applies deep neural networks to secondary structure prediction, where deep neural networks refer to neural networks with more than two hidden layers [44]. To study the disordered regions, the DISOPRED server was used (http://bioinf.cs.ucl.ac.uk/web_servers/disopred/disopred_overview/). This server allows the estimation of the probability of each residue in the sequence of being disordered [45].

### 4.3. Modelling of 3D Structure of hDKC1

The 3D structure predictions (by homology and ab initio) were carried out by the I-TASSER server (available online: http://zhanglab.ccmb.med.umich.edu/I-TASSER/) online database. The hDKC1 sequence was submitted to the online server and the obtained result in PDB format was visualized using Chimera (available online: https://www.cgl.ucsf.edu/chimera). In the prediction of the 3D structure by homology, the protein with PDB ID: 3UAI (crystal structure of Shq1-Cbf5-Nop10-Gar1 complex from *S. cerevisae*) was employed as template.

### 4.4. Validation of the Generated Model

The stereochemical quality of the obtained models was verified using PROCHECK (available online: https://www.ebi.ac.uk/thornton-srv/software/PROCHECK). This software corroborates the quality of a protein structure, producing a number of PostScript plots analyzing its overall and residue-by-residue geometry [12].

### 4.5. Mutation Stability Analysis

By using FoldX (http://foldxsuite.crg.eu), the changes in stability of the mutant hDKC1 (DC reported mutations [6]) were evaluated. The ΔG prediction by Foldx is calculated as the difference in free energy between the unfolded and folded state of the protein. By measuring the difference of unfolding Gibbs energy (ΔΔG) between mutant and wild type, the effect of the mutation on the stability can be calculated [46].

### 4.6. Hydrophobic Pockets Identification

Detection of protein pockets was carried out by FPocket (http://fpocket.sourceforge.net). This software is a quite fast open source protein cavity detection algorithm based on Voronoi tessellations [47].

### 4.7. Docking Based Virtual Screening

To identify potential inhibitors of hDKC1, the homology model of hDKC1 was used. The database employed in the virtual screening was the publicly available Enamine Advanced Collection (available online: https://www.enamine.net). Around 450,000 compounds were screened out of the library. In this study, the residue K314 of the hDKC1 PUA domain was used as a target. The docking was centered in the alpha carbon of K314, with a grid size of 25 Å. This size was defined in order to include all the pockets belonging to PUA domain. AutoDock Vina (https://vina.scripps.edu) was used as docking based virtual screening software. The docking assay for the first hundred compounds was ran one hundred times to determine the docking energy ± SD. The chemical compounds displaying the highest docking scores in the calculations were obtained from Enamine.

### 4.8. Cell Line and Culture Conditions

The human mammary adenocarcinoma cell line MDA MB 231 was obtained from the American Type Culture Collection (ATCC^®^ HTB-26™). Cells were grown in Dulbecco’s modified Eagle’s medium (DMEM) (Thermo Fisher Scientific, Walthman, MA, USA 02451) supplemented with 10% heat-inactivated fetal bovine serum (FBS) (Sigma, St. Louis, MO, USA), 2 mM glutamine and 80 µg/mL gentamicin at 37 °C in 5% CO_2_ atmosphere. Cell cultures were routinely sub-cultured by trypsinization using standard procedures. Cells were treated for 48 h with each compound at a concentration of 10 µM for compounds 1, 2, 3, 4, 5, 6, 7, 8, 9, 12 and 13 and 2 µM for compounds 10 and 15. Dimethyl sulfoxide (DMSO) was used as vehicle.

### 4.9. Determination of Telomerase Activity

Telomerase activity was determined by RQ-TRAP (Real-Time Quantitative Telomerase Repeat Amplification Protocol) assay, using the method with SYBR-Green (StepOne™ System equipment, Thermo Fisher Scientific, Walthman, MA, USA) [48]. Growing tumor cells were harvested and washed once with phosphate buffered saline (PBS). Cells (2 × 10^6^) were transferred to 1.5 mL conical tubes and centrifuged for 8 min at 450 × *g*. The pellet was lysed with 200 µL of CHAPS (3-[(3-Cholamidopropyl)dimethylammonio]-1-propanesulfonate) buffer 0.5% p/v (supplied with RNaseOUT (Thermo Fisher Scientific, Walthman, MA, USA) and a protease inhibitor (Sigma, St. Louis, MO, USA), quantified and stored at −20 °C until use. The RT-qPCR assay was performed in a final volume of 10 µL, using 2 µL of lysate as template, Power SYBR Green Master Mix 1X (Thermo Fisher Scientific, Walthman, MA, USA), ACX primer (Alternative Complementary primer) (250 nM) and TS primer (Telomerase Substrate primer) (800 nM). The PCR cycle started with a 20 min incubation at 25 °C, followed by one step at 90 °C for 10 min and then subjected to 40 PCR cycles at 95°C for 15 s and 60 °C for 10 s. The reaction ended using a melt curve analysis in which the temperature was increased from 55 °C to 95 °C at a linear rate of 0.2 °C/s. Results were analyzed by the StepOne Software v2.3. We evaluated the effect of the compounds on the RQ-TRAP assay. The presence of these compounds at a 10 µM concentration in the reaction mix did not affect the amplification protocol.

## Figures and Tables

**Table 1:** Quality evaluation scores of the predicted 3D structures by I-TASSER.

hDKC1 Model	C-Score	TM Score	RMSD (Å)
Homology Model	−0.09	0.70 ± 0.12	7.1 ± 4.2
Ab initio model	−2.32	0.44 ± 0.14	13.0 ± 4.2

**Table 2:** Analysis of hydrophobic pockets present in the hDKC1 model by FPocket software.

Pocket	Relevance (%)	Volume (Å^3^)	Residue Number
1	100	615	96, 101, 102, 103, 122, 123, 124, 125, 126, 128, 129, 130, 131, 132, 153, 187, 188, 246, 248
2	94	203	299, 300, 301, 314, 315, 317, 320, 322, 323, 350, 354, 355, 358, 360, 361, 362, 363
3	79	284	81, 85, 88, 89, 138, 289, 290, 341, 342, 370, 371, 372, 375
4	69	926	259, 260, 261, 265, 272, 280, 281, 284, 285, 287
5	69	289	141, 142, 144, 145, 293, 296, 332, 333, 334, 344, 345, 346, 367, 368, 370
6	64	810	74, 75, 76, 80, 81, 82, 85, 88, 341, 372, 375
7	60	208	54, 91, 93, 254, 258, 259, 261, 284, 285, 287, 288, 289, 291, 292,
8	60	444	70, 71, 72, 303, 304, 307, 376, 377, 378, 379, 380,
9	58	100	157, 169, 170, 173, 204, 206, 207, 214, 215, 216, 233
10	57	367	98, 127, 129, 154, 155, 156, 215, 245, 246, 247, 249, 256
11	57	994	183, 184, 185, 186, 192, 227, 228, 231, 241, 242, 243
12	54	525	54, 55, 57, 58, 294, 295, 296, 297, 298, 324
13	47	580	301, 302, 304, 305, 308, 309, 313, 314, 315, 316, 318, 319, 378
14	47	158	53, 54, 55, 56, 77, 78, 79, 80, 290, 339, 340
15	44	92	83, 86, 87, 273, 278, 279, 282, 283
16	42	146	176, 180, 181, 182, 197, 199, 218, 220, 224, 225, 226, 228, 229, 232
17	40	295	103, 119, 120, 121, 122, 123, 124, 143, 147, 151, 222, 223
18	37	268	98, 125, 126, 127, 128, 187, 227, 243, 244, 245, 246
19	33	274	156, 206, 207, 208, 211, 213, 214, 215
20	32	425	83, 84, 87, 113, 114, 115, 137, 270, 273, 274, 282
21	28	374	138, 141, 310, 311, 369, 371, 372, 373, 374

**Table 3:** Ranking of compounds obtained from DBVS by AutoDock Vina.

Name	Compound	Docking Energy (kcal/mol)	SD	Name	Compound	Docking Energy (kcal/mol)	SD
E1	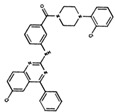	−7.20	0.21	E6	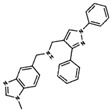	−6.73	0.21
E2	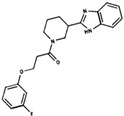	−7.11	0.08	E7	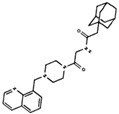	−6.69	0.11
E3	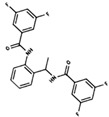	7.04	0.06	E8	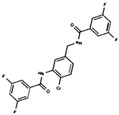	−6.59	0.25
E4	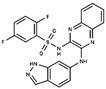	−6.93	0.13	E9	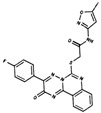	−6.54	0.09
E5	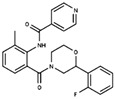	−6.81	0.17	E10	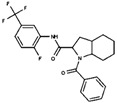	−6.52	0.45

SD = standard deviation.

## References

[1] Alonso D.F., Ripoll G.V., Garona J., Iannucci N.B., Gomez D.E. (2011). Metastasis: Recent discoveries and novel perioperative treatment strategies with particular interest in the hemostatic compound desmopressin. Curr. Pharm. Biotechnol..

[2] Mengual Gomez D.L., Armando R.G., Farina H.G., Gomez D.E. (2014). Telomerase and telomere: Their structure and dynamics in health and disease. Medicina.

[3] Gomez D.E., Armando R.G., Farina H.G., Menna P.L., Cerrudo C.S., Ghiringhelli P.D., Alonso D.F. (2012). Telomere structure and telomerase in health and disease (review). Int. J. Oncol..

[4] Ashbridge B., Orte A., Yeoman J.A., Kirwan M., Vulliamy T., Dokal I., Klenerman D., Balasubramanian S. (2009). Single-molecule analysis of the human telomerase RNA·dyskerin interaction and the effect of dyskeratosis congenita mutations. Biochemistry.

[5] Cerrudo C.S., Ghiringhelli P.D., Gomez D.E. (2014). Protein universe containing a PUA RNA-binding domain. FEBS J..

[6] Angrisani A., Vicidomini R., Turano M., Furia M. (2014). Human dyskerin: Beyond telomeres. Biol. Chem..

[7] Garofola C., Gross G.P. (2018). Dyskeratosis Congenita.

[8] Dokal I. (2011). Dyskeratosis congenita. Hematology ASH Education Program.

[9] Zeng X.L., Thumati N.R., Fleisig H.B., Hukezalie K.R., Savage S.A., Giri N., Alter B.P., Wong J.M. (2012). The accumulation and not the specific activity of telomerase ribonucleoprotein determines telomere maintenance deficiency in X-linked dyskeratosis congenita. Hum. Mol. Genet..

[10] Arndt G.M., MacKenzie K.L. (2016). New prospects for targeting telomerase beyond the telomere. Nat. Rev. Cancer.

[11] Zhang Y. (2008). I-TASSER server for protein 3D structure prediction. BMC Bioinform..

[12] Laskowski R.A., Rullmannn J.A., MacArthur M.W., Kaptein R., Thornton J.M. (1996). AQUA and PROCHECK-NMR: Programs for checking the quality of protein structures solved by NMR. J. Biomol. NMR.

[13] Bromberg Y., Rost B. (2009). Correlating protein function and stability through the analysis of single amino acid substitutions. BMC Bioinform..

[14] Wang C., Xu P., Zhang L., Huang J., Zhu K., Luo C. (2018). Current Strategies and Applications for Precision Drug Design. Front. Pharmacol..

[15] Singla R.K. (2015). Editorial: In silico drug design and medicinal chemistry. Curr. Top. Med. Chem..

[16] Zheng M., Zhao J., Cui C., Fu Z., Li X., Liu X., Ding X., Tan X., Li F., Luo X. (2018). Computational chemical biology and drug design: Facilitating protein structure, function, and modulation studies. Med. Res. Rev..

[17] Cardama G.A., Comin M.J., Hornos L., Gonzalez N., Defelipe L., Turjanski A.G., Alonso D.F., Gomez D.E., Menna P.L. (2014). Preclinical development of novel Rac1-GEF signaling inhibitors using a rational design approach in highly aggressive breast cancer cell lines. Anticancer Agents Med. Chem..

[18] Li S., Duan J., Li D., Ma S., Ye K. (2011). Structure of the Shq1-Cbf5-Nop10-Gar1 complex and implications for H/ACA RNP biogenesis and dyskeratosis congenita. EMBO J..

[19] Singh M., Wang Z., Cascio D., Feigon J. (2015). Structure and interactions of the CS domain of human H/ACA RNP assembly protein Shq1. J. Mol. Biol..

[20] Rashid R., Liang B., Baker D.L., Youssef O.A., He Y., Phipps K., Terns R.M., Terns M.P., Li H. (2006). Crystal structure of a Cbf5-Nop10-Gar1 complex and implications in RNA-guided pseudouridylation and dyskeratosis congenita. Mol. Cell.

[21] Li L., Ye K. (2006). Crystal structure of an H/ACA box ribonucleoprotein particle. Nature.

[22] Zhou H., Skolnick J. (2007). Ab initio protein structure prediction using chunk-TASSER. Biophys. J..

[23] Moult J., Pedersen J.T., Judson R., Fidelis K. (1995). A large-scale experiment to assess protein structure prediction methods. Proteins.

[24] Cozzetto D., Kryshtafovych A., Tramontano A. (2009). Evaluation of CASP8 model quality predictions. Proteins.

[25] Abdelmonsef A.H., Dulapalli R., Dasari T., Padmarao L.S., Mukkera T., Vuruputuri U. (2016). Identification of Novel Antagonists for Rab38 Protein by Homology Modeling and Virtual Screening. Comb. Chem. High Throughput Screen..

[26] Kanwal S., Jamil F., Ali A., Sehgal S.A. (2017). Comparative Modeling, Molecular Docking, and Revealing of Potential Binding Pockets of RASSF2; a Candidate Cancer Gene. Interdiscip. Sci..

[27] Rout S., Mahapatra R.K. (2016). In silico screening of novel inhibitors of M17 Leucine Amino Peptidase (LAP) of *Plasmodium vivax* as therapeutic candidate. Biomed. Pharmacother..

[28] Rashidieh B., Madani Z., Azam M.K., Maklavani S.K., Akbari N.R., Tavakoli S., Rigi G. (2015). Molecular docking based virtual screening of compounds for inhibiting sortase A in *L. monocytogenes*. Bioinformation.

[29] Aruleba R.T., Adekiya T.A., Oyinloye B.E., Kappo A.P. (2018). Structural Studies of Predicted Ligand Binding Sites and Molecular Docking Analysis of Slc2a4 as a Therapeutic Target for the Treatment of Cancer. Int. J. Mol. Sci..

[30] Adekiya T.A., Aruleba R.T., Khanyile S., Masamba P., Oyinloye B.E., Kappo A.P. (2017). Structural Analysis and Epitope Prediction of MHC Class-1-Chain Related Protein-A for Cancer Vaccine Development. Vaccines (Basel).

[31] Yu Y.T., Meier U.T. (2014). RNA-guided isomerization of uridine to pseudouridine--pseudouridylation. RNA Biol..

[32] Savage S.A., Adam M.P., Ardinger H.H., Pagon R.A., Wallace S.E., Bean L.J.H., Stephens K., Amemiya A. (1993). Dyskeratosis Congenita. GeneReviews^®^.

[33] Halim S.A., Khan S., Khan A., Wadood A., Mabood F., Hussain J., Al-Harrasi A. (2017). Targeting Dengue Virus NS-3 Helicase by Ligand based Pharmacophore Modeling and Structure based Virtual Screening. Front. Chem..

[34] Cardama G.A., Gonzalez N., Maggio J., Menna P.L., Gomez D.E. (2017). Rho GTPases as therapeutic targets in cancer (Review). Int. J. Oncol..

[35] Gentile F., Barakat K.H., Tuszynski J.A. (2018). Computational Characterization of Small Molecules Binding to the Human XPF Active Site and Virtual Screening to Identify Potential New DNA Repair Inhibitors Targeting the ERCC1-XPF Endonuclease. Int. J. Mol. Sci..

[36] Lipinski C.A., Lombardo F., Dominy B.W., Feeney P.J. (2001). Experimental and computational approaches to estimate solubility and permeability in drug discovery and development settings. Adv. Drug Deliv. Rev..

[37] Cheng T., Li Q., Zhou Z., Wang Y., Bryant S.H. (2012). Structure-based virtual screening for drug discovery: A problem-centric review. AAPS J..

[38] Chiba S., Ishida T., Ikeda K., Mochizuki M., Teramoto R., Taguchi Y.H., Iwadate M., Umeyama H., Ramakrishnan C., Thangakani A.M. (2017). An iterative compound screening contest method for identifying target protein inhibitors using the tyrosine-protein kinase Yes. Sci. Rep..

[39] Kolosenko I., Yu Y., Busker S., Dyczynski M., Liu J., Haraldsson M., Palm Apergi C., Helleday T., Tamm K.P., Page B.D.G. (2017). Identification of novel small molecules that inhibit STAT3-dependent transcription and function. PLoS ONE.

[40] Billones J.B., Carrillo M.C., Organo V.G., Sy J.B., Clavio N.A., Macalino S.J., Emnacen I.A., Lee A.P., Ko P.K., Concepcion G.P. (2017). In silico discovery and in vitro activity of inhibitors against Mycobacterium tuberculosis 7,8-diaminopelargonic acid synthase (Mtb BioA). Drug Des. Dev. Ther..

[41] Du H., Brender J.R., Zhang J., Zhang Y. (2015). Protein structure prediction provides comparable performance to crystallographic structures in docking-based virtual screening. Methods.

[42] Tarazi H., Saleh E., El-Awady R. (2016). In-silico screening for DNA-dependent protein kinase (DNA-PK) inhibitors: Combined homology modeling, docking, molecular dynamic study followed by biological investigation. Biomed. Pharmacother..

[43] Kumar Deokar H., Barch H.P., Buolamwini J.K. (2017). Homology Modeling of Human Concentrative Nucleoside Transporters (hCNTs) and Validation by Virtual Screening and Experimental Testing to Identify Novel hCNT1 Inhibitors. Drug Des..

[44] Heffernan R., Dehzangi A., Lyons J., Paliwal K., Sharma A., Wang J., Sattar A., Zhou Y., Yang Y. (2016). Highly accurate sequence-based prediction of half-sphere exposures of amino acid residues in proteins. Bioinformatics.

[45] Ward J.J., McGuffin L.J., Bryson K., Buxton B.F., Jones D.T. (2004). The DISOPRED server for the prediction of protein disorder. Bioinformatics.

[46] Li L., Ye Y., Sang P., Yin Y., Hu W., Wang J., Zhang C., Li D., Wan W., Li R. (2017). Effect of R119G Mutation on Human P5CR1 Dynamic Property and Enzymatic Activity. Biomed. Res. Int..

[47] Le Guilloux V., Schmidtke P., Tuffery P. (2009). Fpocket: An open source platform for ligand pocket detection. BMC Bioinform..

[48] Wege H., Chui M.S., Le H.T., Tran J.M., Zern M.A. (2003). SYBR Green real-time telomeric repeat amplification protocol for the rapid quantification of telomerase activity. Nucleic Acids Res..

